# A Perspective on Removal of Cyanotoxins from Water Through Advanced Oxidation Processes

**DOI:** 10.1002/gch2.202300125

**Published:** 2023-09-01

**Authors:** Shilpi Verma, Praveen Kumar, Urška Lavrenčič Štangar

**Affiliations:** ^1^ School of Energy & Environment Thapar Institute of Engineering & Technology Patiala Punjab 147004 India; ^2^ Faculty of Chemistry and Chemical Technology University of Ljubljana Ljubljana 1000 Slovenia

**Keywords:** advanced oxidation processes, cyanotoxins, sulfate radicals

## Abstract

This perspective discusses the challenges associated with the removal of cyanotoxins from raw water sources for drinking water treatment and the emergence of sulfate radical‐based advanced oxidation processes (SR‐AOPs) as an effective treatment technique. The advantage of SR‐AOPs is that they can be activated using a variety of methods, including heat, UV radiation, and transition metal catalysts, allowing for greater flexibility in treatment design and optimization. In addition, the byproducts of SR‐AOPs are less harmful than those generated by ^•^OH‐AOPs, which reduces the risk of secondary contamination. SR‐AOPs generate sulfate radicals (SO_4_^•−^) that are highly selective to certain organic contaminants and have lower reactivity to background water constituents, resulting in higher efficiency and selectivity of the process. The presence of natural organic matter and transition metals in the natural water body increases the degradation efficiency of SR‐AOPs for the cyanotoxins. The bromate formation is also suppressed when the water contaminated with cyanotoxins is treated with SR‐AOPs.

## Graphical abstract

Sulfate radical‐based advanced oxidation processes (AOPs) excel in cyanotoxin removal. Their high reactivity efficiently degrades diverse cyanotoxins, ensuring safe water quality. Their versatility and effectiveness make them the most suitable AOPs and safeguard aquatic ecosystems and human health.

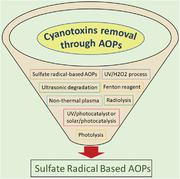

## 1 Introduction

Global warming and water pollution from nutrients have accelerated the occurrence of algal blooms in drinking water sources.^[^ ^1^, ^2^ ^]^ Algal blooms in aquatic ecosystems can pose a serious threat to the ecosystem. The effects of excessive algal growth on the environment include a decrease in dissolved oxygen, the sequestration of nutrients by algae, which reduces the amount of nutrients available to other species, and the absorption of sunlight by the blooming algal biomass, which decreases light penetration necessary for other species.^[^ ^3^, ^4^ ^]^

An overgrowth of cyanobacteria or blue–green algae is referred to as a “cyanobacterial bloom.” Cyanobacteria or blue–green algae are prokaryotic photoautotrophic organisms. Cyanobacterial blooms are usually blue–green in color but can be other colors, such as red or brown.^[^ ^5^ ^]^ Forty of the 150 genera of cyanobacteria produce toxic secondary metabolites. These toxic groups of secondary metabolites are known as cyanotoxins.^[^ ^4^, ^6^, ^7^ ^]^ The occurrence of toxic cyanobacteria has been reported from various geographic locations around the world including Europe, the United States, Asia, Middle East, Australia, Africa, and Antarctica.^[^ ^4^, ^8^, ^9^, ^10^ ^]^ Since 1985, reports of harmful algal events that impact on human society have been collected in the Harmful Algae Event Database, HAEDAT.^[^ ^11^ ^]^ The 2019 Intergovernmental Panel for Climate Change (IPCC) assessment report suggests that there will be a continued increase in the occurrence, toxicity, and risk of harmful algal blooms (HABs) on both marine and freshwater systems in the 21st century due to the effects of global warming and rising levels of carbon dioxide.^[^ ^12^ ^]^

**Figure 1:**
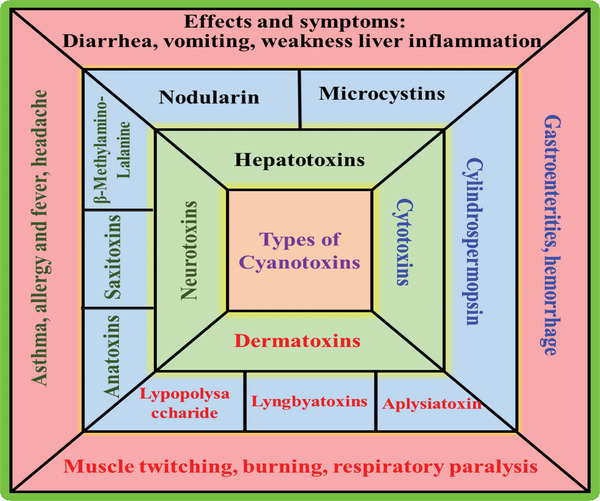
Types and effects of harmful cyanotoxins involved in lethal algal bloom events.

Cyanotoxins can be divided into three groups based on their chemical structure: lipopolysaccharides (LPS), cyclic peptides (hepatotoxins), and alkaloids (neurotoxins).^[^ ^4^, ^7^ ^]^ Another way to classify cyanotoxins is based on the function of the organs affected by cyanotoxins (**Figure** **1**), e.g., hepatotoxins (such as anatoxin, saxitoxin, and microcystin), neurotoxins (such as nodularin, and cylindrospermopsin), and dermatotoxins (e.g., aplysiatoxin, lyngbyatoxin‐A).^[^ ^13^, ^14^ ^]^

Over time, several of these cyanotoxins have also been shown to be carcinogenic.^[^ ^14^, ^15^ ^]^ Cyanotoxins can be present either inside the undamaged cyanobacterial cell, which is referred to as “intracellular” or “cell‐bound,” or outside the cell in the aqueous environment, which is referred to as “extracellular” or “dissolved.” The exact reason why an algal bloom releases or does not release toxins is unclear. Different strains of the same species may lack the gene cluster responsible for toxin production or have it in an inactive state that prevents toxin synthesis.^[^ ^16^ ^]^ In strains capable of toxin production, intracellular toxin content has been found to be modulated by a variety of environmental or physiological factors such as temperature, light, nutrient availability, presence of heavy metals, hydrodynamic conditions, organic material, cell density, and grazing by predators.^[^ ^17^, ^18^ ^]^ Given the complexity of interactions among these different environmental triggers, predicting toxic production in HAB is still a major challenge.^[^ ^19^ ^]^

**Figure 2:**
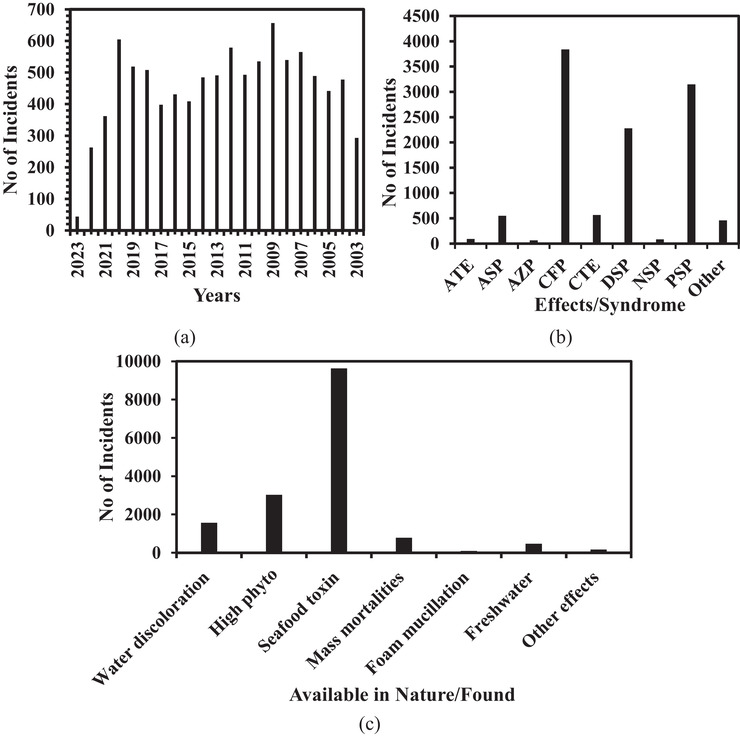
a) Harmful algal incidents occurred in the last two decades. b) Effect versus incidents aerosolized toxins effects (ATE); amnesic shellfish poisoning (ASP); azaspiracid shellfish poisoning (AZP); ciguatera fish poisoning (CFP); cyanobacterial toxins effects (CTE); diarrhetic (diarrheic) shellfish poisoning (DSP); neurotoxic shellfish poisoning (NSP); paralytic shellfish poisoning (PSP); Other. c) Nature of harmful algal events.

The cyanotoxins are responsible for intermittent but repeated widespread poisoning of wild and domestic animals and aqua cultured fish. The first documented fatal poisoning of humans by cyanobacterial hepatotoxins, via the intravenous route, was reported in 1996 at a dialysis clinic in the city of Caruaru, Brazil.^[^ ^19^, ^20^ ^]^ In the last two decades, a high number of human and animal poisoning cases have been reported worldwide,^[^ ^21^ ^]^ as shown in **Figure** **2**.

The cyanotoxins responsible for the poisonings were microcystins (MC), nodularins, cylindrospermopsins (CYN), anatoxins (ATX), and saxitoxins (STX).^[^ ^20^ ^]^ Therefore, reliable monitoring and treatment technologies are essential to prevent the adverse effects of cyanotoxins in drinking water sources.

Depending on the cyanotoxin under consideration, monitoring of cyanotoxins can be based on a variety of immunological, biological, or physicochemical methods.^[^ ^22^, ^23^ ^]^ The low detection limit of some analytical techniques for cyanotoxins, the high cost of analysis, and the co‐occurrence of different cyanotoxins with multiple mechanisms of action that cannot all be detected by the same biological or immunochemical assay make the detection of cyanotoxins challenging.^[^ ^14^, ^18^, ^24^, ^25^ ^]^ Compared to other types of water contaminants, such as pharmaceuticals, hormones, or pesticides, the scientific community has paid relatively little attention to the removal of cyanotoxins. However, it is critical to address cyanotoxin pollution because it poses a significant public health risk due to its extremely high toxicity and the increasing frequency of cyanobacterial blooms affecting drinking water sources. One possible explanation for the lack of research in this area is the high cost of purified cyanotoxins, the need for sensitive analytical equipment to quantify them, and the difficulty of obtaining samples from natural water sources.^[^ ^26^ ^]^

Water treatment plants face several unique challenges in removing or degrading cyanotoxins from water sources.^[^ ^27^ ^]^ Some of these challenges include:

a) Variability in cyanotoxin concentrations and types: Concentrations and types of cyanotoxins can vary widely depending on the type of cyanobacteria present, the time of year, and environmental conditions. This variability can make it difficult to develop effective treatment strategies that can handle a range of cyanotoxin types and concentrations.
b) Limited treatment options: Many conventional water treatment processes, such as coagulation, flocculation, sedimentation, and filtration, are not effective in removing extracellular/ dissolved cyanotoxins. Some cyanotoxins are also resistant to conventional disinfection methods, such as chlorination via the usage of chlorine dioxide, chlorine, and chloramine.
c) Cyanobacteria cell disruption: In some cases, preoxidation processes such as the use of potassium permanganate, which effectively removes cyanobacteria may also result in the release of intracellular cyanotoxins into the water, making their removal more difficult.
d) Cost and complexity of treatment: Some treatment options, such as activated carbon adsorption, advanced oxidation processes, and membrane filtration, can effectively remove cyanotoxins, but are expensive and require specialized equipment and expertise.
e) Potential for toxin regrowth: Even if cyanotoxins are effectively removed or degraded, there is always the possibility that new blooms will occur and toxin levels will rise again. This requires continuous monitoring and treatment to ensure safe drinking water.

## 2 Regulations to Control Exposure to Cyanotoxins

National and international advisories, recommendations, and regulations for cyanotoxin levels in drinking and recreational water bodies are becoming more prevalent. A provisional guideline value (GV) established by the World Health Organization (WHO) for MC‐LR is for lifetime drinking water GV: 1 µg L^−1^; for short‐term drinking‐water GV: 12 µg L^−1^ and for recreational waters GV: 24 µg L^−1^. However, no guidelines have been published for other MC variants or other cyanotoxins.^[^ ^28^ ^]^ While Australia established standards of 1.3 µg L^−1^ for total MCs and published health advisories of 1 and 3 µg L^−1^ for CYNs and saxitoxins, respectively, other countries, such as Canada, have set upper limits of 1.5 µg ^−1^L for total MCs. This information has supported drinking water recommendations, guidelines, and restrictions in countries such as New Zealand, Uruguay, Singapore, France, and the Czech Republic. The EU Drinking Water Directive (Council Directive 98/83/EC and 2015/1787 on the quality of water intended for human consumption) addresses the quality of water intended for human consumption. Algal toxins released during algal blooms are listed in S40 ALGALTOX on the NORMAN Suspect List Exchange.^[^ ^29^ ^]^ Cyanotoxins are not subject to formal regulation in the U.S., although anatoxin‐a, cylindrospermopsin, microcystins, and saxitoxin are listed on the U.S. Environmental Protection Agency's Candidate Contaminant List 4 of the US (EPA).^[^ ^30^ ^]^ According to WHO, existing cyanotoxin safety recommendations are unacceptable for use in recreational waters. Based on cyanobacterial cell density, biovolume, and pigment content—all of which closely correlate with concentrations of cyanobacteria and cyanotoxins—WHO has proposed standards and regulations. In most circumstances, countries establish two‐ or three‐level alert levels to indicate the potential risk to human health based on these factors.^[^ ^31^ ^]^ Improving available toxicological data that can lead to the establishment of more informed limits for cyanotoxins in drinking water and developing more accurate and rapid monitoring and analytical methods are urgently needed to advance the regulation of cyanotoxins.^[^ ^14^ ^]^

## 3 Removal of Cyanotoxins from the Water

**Figure 3:**
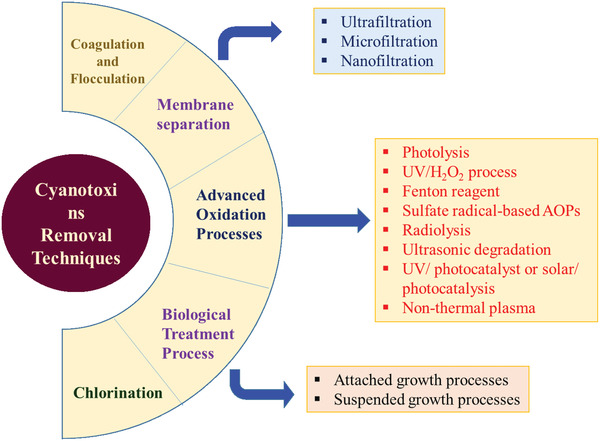
Cyanotoxins removal techniques.

To protect human and animal life, the first strategy should focus on preventing the development of cyanobacterial blooms in surface waters through measures, such as nitrogen reduction, biomanipulation, or the use of algaecides. Removal of intact cyanobacterial cells is critical, especially in the case of toxic cyanobacteria, to prevent the release of internal toxins.^[^ ^32^ ^]^ The second strategy involves the elimination of both cyanobacterial cells and metabolites, i.e., cyanotoxins in water treatment plants. Most traditional drinking water treatment processes successfully eliminate cyanobacterial cells and intracellular metabolites. However, the extracellular and dissolved cyanotoxins can escape conventional techniques, such as fast sand filtration and coagulation (**Figure** **3**).^[^ ^33^, ^34^ ^]^ Removal of dissolved cyanotoxins from water can be even more difficult than removal of suspended or adherent cyanotoxins because they are often present at very low concentrations and are difficult to detect and remove. Removal requires careful consideration of the type of cyanotoxin present, the specific treatment method required, and the cost and feasibility of the treatment method. Although conventional treatment methods, such as physical containment, biodegradation, or chemical oxidation can be successful, they all have a number of practical, economic, or environmental drawbacks.^[^ ^33^ ^]^

Physical retention can eliminate toxins by using filter membranes with extremely small pore sizes, such as nanofiltration and reverse osmosis.^[^ ^33^, ^35^ ^]^ The concentrate stream generated by reverse osmosis (RO) and nanofiltration processes has the potential to retain a high level of cyanotoxins, as these processes are capable of eliminating extracellular/dissolved cyanotoxins. In addition, it may be necessary to backwash filter beds and membranes frequently to prevent clogging, fouling, and cyanobacterial growth on the filter media. It is imperative to consider the potential residual disposal issues that may arise due to high concentrations of cyanotoxins in the concentrate stream and the backwash water stream.^[^ ^33^, ^35^, ^36^ ^]^

Activated carbon, either in the form of powdered activated carbon (PAC) or granular activated carbon (GAC), is commonly used in water treatment plants to remove unpleasant taste and odor (T&O) and other organic compounds by the adsorption process. Recent studies have shown that the use of PAC or GAC is effective in removing microcystins from water.^[^ ^37^, ^38^ ^]^ Limited studies have also shown promising results in the removal of cylindrospermopsin, anatoxin‐a, and saxitoxin. The effectiveness of carbon adsorption in removing extracellular cyanotoxins depends on the type of carbon and its corresponding mesopore size. The presence of other organic compounds such as humic acid or minerals in the water may interfere with the adsorption process and reduce the effectiveness of the activated carbon. Therefore, it is important to carefully evaluate the specific conditions and types of cyanotoxins present in the water before relying solely on activated carbon for removal.^[^ ^39^ ^]^

Although some cyanotoxins can be biodegraded, their repeated occurrence may limit the ability of microorganisms to do so, resulting in an initial lag‐phase of up to a few days without precondition.^[^ ^33^, ^40^ ^]^ It is difficult to estimate the efficacy of a biological treatment barrier and potential drawbacks, such as biotransformation of the less toxic cyanotoxins into the more toxic substances, as most enzymatic degradation mechanisms are still poorly understood.^[^ ^33^, ^35^, ^40^ ^]^

While permanganate and chlorine are effective oxidants for the degradation of some cyanotoxins, others are either resistant or require much higher oxidant concentrations and reaction times than those normally used in drinking water treatment.^[^ ^33^, ^41^ ^]^ Studies have shown that potassium permanganate (KMnO_4_) is a relatively effective treatment method for microcystin‐LR and anatoxin‐a, although treatment efficacy is independent of pH, for microcystins, while it is dependent on pH for anatoxin‐a. However, this method has been shown to be ineffective in removing cylindrospermopsin. Dosage also plays an important role. A higher than optimal dose results in the release of intracellular cyanotoxin into the water due to cell lysis.^[^ ^39^, ^42^ ^]^

A major disadvantage of chlorination is the production of halogenated disinfection by‐products resulting from the reaction between chlorine and organic material or bromide in the water. In addition, residual chlorine can negatively affect drinking water quality due to its potentially objectionable taste and odor.^[^ ^33^, ^43^ ^]^

Advanced oxidation processes (AOPs) have attracted considerable attention in drinking water and wastewater treatment due to their effectiveness in degrading even poorly degradable organic compounds and disinfecting pathogens. AOPs are an alternative type of oxidation that has been extensively researched and applied. By producing radicals, AOPs are able to degrade cyanotoxins. Depending on the AOP process, these radicals can be either hydroxyl or sulfate radicals.^[^ ^44^, ^45^ ^]^ AOPs involving hydrogen peroxide, ozone, photolysis (including the use of oxidants and catalysts), Fenton oxidation, nonthermal plasmas, sulfate radicals, electrochemical oxidation, sonolysis, and radiolysis have been used as sources for the generation of hydroxyl or sulfate radicals.^[^ ^46^ ^]^ As shown in Figure 1, the use of AOPs for the treatment of water contaminated with cyanotoxins has increased in the last two decades. Degradation efficiency is one of the most important factors to study when comparing different AOPs, especially when considering their use in large‐scale water treatment. It establishes a link between the amount of energy, oxidant, or catalyst required and the effectiveness of the treatment. Electrical energy per order (*E*_EO_) is often chosen as a metric for the energy efficiency of AOPs.^[^ ^46^ ^]^ For zero order kinetics “electrical energy per mass (*E*_EM_(kWh kg^−1^))” and for first order kinetics “electrical energy per order (*E*_EO_)(kWh m^−3^order^−1^))” are often chosen as performance metrics for the energy efficiency of AOPs. Such performance metrics are useful not only to compare AOPs, but also to provide the necessary data for scale‐up and economic evaluations in contrast to conventional wastewater treatment technologies. Cyanotoxin removal efficiency by AOPs has been reported to be in the range of 10–100% under various operating conditions.^[^ ^14^, ^33^ ^]^

Hydroxyl radical‐based AOPs (^•^OH‐AOPs) use hydroxyl radicals (^•^OH) to oxidize pollutants. Hydroxyl radical‐based AOPs such as photo‐Fenton, the Fenton process, ozone‐based AOPs, electrochemical oxidation, plasma‐based AOPs, photocatalysis, and sonolysis are effective in removing a variety of organic pollutants from the various industrial and municipal wastewater streams. These hydroxyl radicals are highly reactive and nonselective. As a result, they can react with a wide range of organic and inorganic compounds.^[^ ^47^ ^]^ However, the nonselective nature of hydroxyl radicals also makes them susceptible to scavenging by matrix constituents, such as natural and organic matter from effluents (NOM/EfOM) and common anions. In recent years, there has been increased interest in the use of other reactive species for the oxidation of organic contaminants in aqueous environments, some of them are less affected by NOM or EfOM. One such reactive species is the sulfate radical, which has shown promise for the effective degradation of organic contaminants in the presence of NOM/EfOM.^[^ ^48^ ^]^

### 3.1 Sulfate Radical‐Based AOP (SR‐AOP)

Sulfate radical‐based AOPs (SO_4_^•−^‐AOP) have attracted significant interest in recent decades as a viable alternative to conventional hydroxyl radical‐based AOPs (^•^OH‐ AOPs) in water and wastewater treatment to overcome the technical constraints of H_2_O_2_. The first reason for the significant interest of the scientific community in SO_4_^•−^‐AOP was its redox potential. The redox potential of sulfate radicals is higher than that of hydroxyl radicals, namely *E* ^0^(SO_4_^•−^/SO_4_^2−^) = +2.60 – +3.10 VNHE > *E* ^0^(^•^OH/OH^−^) = +1.90 – +2.70 VNHE. Other reasons are the higher yield of radical formation that can be achieved, the lesser influences of operating factors, the involvement of various nonradical and radical species in the degradation/oxidation, and various persulfate activation techniques.^[^ ^49^ ^]^ The optimism that currently permeates academic research is also reflected in the rise in scientific papers, most of which focus on the development of persulfate activation techniques. Although SR‐AOPs have demonstrated success in the applications cited earlier, like many AOPs, their real‐world applications are relatively limited in comparison to the extensive scientific literature and laboratory‐scale tests. the limited availability of suitable reactors for the implementation of SR‐AOPs poses a significant obstacle to their widespread utilization.^[^ ^44^ ^]^ AOPs with sulfate radicals (SR‐AOPs) and AOPs with hydroxyl radicals (^•^OH‐AOPs), both are used to oxidize pollutants in water and wastewater treatment.^[^ ^50^, ^51^, ^52^, ^53^ ^]^ However, the mechanisms and chemistry of SR‐AOPs differ significantly from those of ^•^OH‐AOPs. Neta et al.^[^ ^54^ ^]^ reported that sulfate radicals are more efficient oxidants for the removal of organic compounds with unsaturated bonds and aromatic components than hydroxyl radicals due to their selectivity.

The commonly used salts in SR‐AOPs are PMS (peroxymonosulfate; HSO_5_^−^) and PDS (peroxydisulfate; S_2_O_8_^2−^). PDS and PMS can be activated by a variety of methods, including heat, UV, alkyl, metal ions, and activated carbon.^[^ ^55^, ^56^ ^]^ In addition, PS and PMS are more stable than H_2_O_2_, allowing precursors to move over longer distances in water.^[^ ^57^, ^58^ ^]^ Once activated, persulfate or peroxydisulfate can generate a variety of reactive species, including sulfate radicals (SO_4_^•−^), singlet oxygen (^1^O_2_), ^•^OH, and O_2_^•−^, as shown in **Table** **1**. Unlike ^•^OH, SO_4_^•−^ are relatively selective, i.e., they tend to react with certain types of organic compounds, such as alcohols, aldehydes, and ketones. Singlet oxygen is less reactive than ^•^OH radicals and SO_4_^•−^, but can still oxidize certain types of organic compounds.^[^ ^49^, ^56^ ^]^

**Table 1:** It illustrates how the activation method influences the generation of reactive species from PMS and PDS/ PS

Activated method	Oxidative species	
	PMS (HSO_5_^−^) 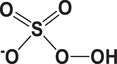	PDS (S_2_O_8_^2−^) 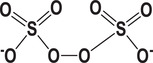
Heat	SO_4_^•−^, HO^•^	SO_4_^•−^
Alkali	O_2_^•−^, ^1^O_2_	SO_4_^•−^
Radiation	SO_4_^•−^, HO^•^	SO_4_^•−^
Transition metal	SO_4_^•−^, SO_5_^−^	SO_4_^•−^
Carbonaceous materials	SO_4_^•−^	SO_4_^•−^
Phenol/quinones	^1^O_2_	SO_4_^•−^

**Figure 4:**
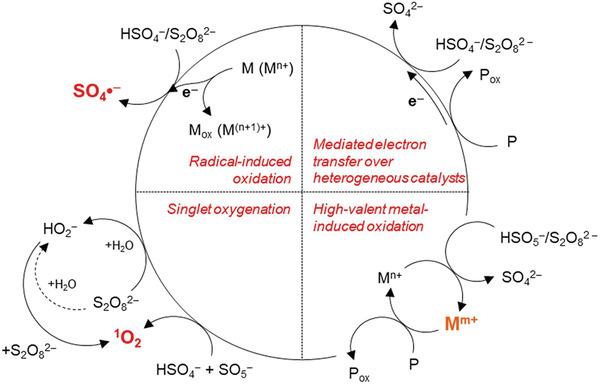
Potential pathways of oxidative reactions produced by the activation of PMS and PDS. Reproduced with permission.^[^ ^49^ ^]^ Copyright 2020, American Chemical Society.

SO_4_^•−^ generally undergoes three different reaction pathways (**Figure** **4**) with organic pollutants: i) hydrogen abstraction from C─H bonds, ii) addition to unsaturated bonds, and iii) electron transfer reactions from carboxylates, amines, and aromatic compounds.^[^ ^49^, ^59^, ^60^ ^]^ The third mechanism facilitates decarboxylation, which, except for SR‐AOPs, has been observed only in the UV‐based degradation of cyanotoxins.^[^ ^33^, ^58^, ^61^ ^]^ For the removal of cyanotoxins from water, SR‐AOPs were mainly studied for the removal of ATX, MCs, and CYN, focusing on UV and catalyst activation as shown in **Table** **2**.^[^ ^62^, ^63^, ^64^, ^65^, ^66^, ^67^, ^68^, ^69^, ^70^, ^71^, ^72^, ^73^, ^74^, ^75^ ^]^

**Table 2:** Degradation of cyanotoxins through various AOPs

Technique	Cyanotoxins	Reaction conditions	Removal	Refs.
NF‐TiO_2_	Microcystin‐LR, ‐RR, ‐LA, ‐YR, and CYN	pH = 3.0, NOM conc = 5–10 mg L^−1^, HCO_3_^−^ and CO_3_^2−^ = 50–150 mg L^−1^,	100% removal in 2 h	[62]
(TiO_2_/ZnO)	Microcystin‐LR	pH = 6, amount of photocatalyst = 50 mg, Conc of MC‐LR = 50 µg L^−1^	100% removal in 5 min	[63]
UV‐C LEDs/UV‐C LED/H_2_O_2_	Anatoxin‐a	UV fluence 4032 J m^−2^. The optimum reaction conditions were: *λ* = 260 nm, initial pH = 6.4, temperature = 24 °C and distance from water surface = 5 mm.	Removal = 50% with UV‐C LEDs and 97% with UV‐C LED/H_2_O_2_	[64]
UV LED/ TiO_2_ coated hollow glass spheres	Nodularin and 11 microcystin variants	Different catalyst loads (0.2; 0.4; 0.6%; 0.8%), light source–LED and Xenon lamp	All the cyanotoxins were removed in less than 5 min	[65]
UV/TiO_2_/HiO_2_	Microcystin‐LR	pH 3.5, 0.05 g L^−1^ TiO_2_, and 0.05 mmol L^−1^ H_2_O_2_, MC‐LR (308 µg L^−1^)	Microcysntin‐LR was completely degraded within 60 min	[66]
C‐TiO_2_/VIS	Microcystin‐LR and Cylindrospermopsin	MC‐LR and CYN (10 mg L^−1^ for UV‐A and 2 mg L^−1^ for VIS), amount of C‐TiO_2_/VIS 200 mg L^−1^, temp = 25 °C,	C‐TiO2/VIS was found very effective in degrading MC‐LR and CYN	[67]
PS and PMS coupled with UVA/TiO	Microcystin‐LR	Conc of MC‐LR = 5 mg L^−1^, pH = 5.6, temp = 32 °C, Conc of PMS or PS = 0.0208 mm, TiO_2_ 10 mg L^−1^	Degradation efficiency of the treatment techniques: UVA/TiO_2_ < UVA/TiO_2_/PS (52%) < UVA/TiO_2_/PMS (77%).	[68]
Fe_2_O_3_ nanopowder/ visible light	Microcystin‐LR	MC‐LR = 500 µg L^−1^, pH = 3–10, amount of Fe_2_O_3_ = 0.5 g L^−1^	86% removal was observed	[69]
RuO_2_‐TiO_2_/Ti and stainless steel as anode and cathode with both UV365 or UV254 irradiation	Microcystin‐LR	Conc. MC‐LR = 76.1–328.7 µg L^−1^, Electrolytes: NaCl, Na_2_SO_4_, Na_2_HPO_4_ and NaNO_3_;	With 0.02 mol L^−1^ NaCl, 100% removal was observed	[70]
TiO_2_ coated glass beads/UV	Four variants of Microcystin‐LR, ‐LY, ‐LW, ‐LF	Stainless steel photo‐reactors were used with maintained temperature	92% removal of four microcystins after UV‐A photolysis	[71]
FeIII‐B*/H_2_O_2_	Cylindrospermopsin and anatoxin‐a	NOM conc = 0–30 ppm, pH = 8.5–11.5,	CYL removed by 93% and ANA by 88% after 2 h	[72]
Fe^2+^/H_2_O_2_, Co_2_^+^/PMS, Ag^+^/PS	Cylindrospermopsin	Conc. of CYN = 10 µm, Fe_2_+ = 200 µm, H_2_O_2_ = 100 µm, Co_2_+ = 40 µm, PMS = 80 µm	Within 15 min, 98% CYN was degraded	[73]
Ferrate (VI)	Cylindrospermopsin (CYN)	Conc of CYN = 2.0 µm, Fe(VI) = 50 µm, pH = 7–9.5	CYN can be completely degraded by within 2 h	[74]
C_3_N_5_ nanosheet	Microcystin‐LR	Conc. of MC‐LR = 4 mg L^−1^, pH = natural	99.99% degradation of MC‐LR at 45 min	[75]

#### 3.1.1 Degradation Mechanism of CYN through SR‐AOP

**Figure 5:**
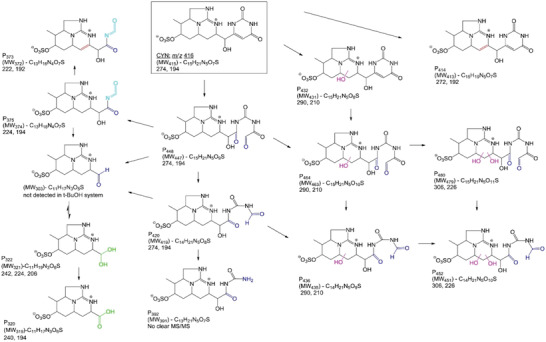
Main degradation pathways of cylindrospermopsin by SR‐AOPs. Reproduced with permission.^[^ ^77^ ^]^ Copyright 2014, Elsevier.

It was observed that in the case of CYN, UV‐254 nm radiation did not lead to significant degradation. However, UV/PMS and UV/ PDS or PS significantly enhanced the degradation of CYN with a second‐order rate constant k_SO4_^•−^ /_CYN_ of 4.5 × 10^9^ m ^−1^ s^−1^. Transition metals present in tap water were found to contribute to the degradation of CYN in the UV/PMS and UV/PS systems. However, the presence of natural organic matter (NOM) did not significantly hinder the degradation of CYN.^[^ ^76^ ^]^ The attack of the sulfate radical on the tricyclic alkaloid group of CYN most likely occurs through a weak H‐abstraction leading to selective hydroxylation and dehydrogenation. The sulfate radical reaction at the tricyclic alkaloid group most likely occurred by weak H‐abstraction during the degradation of CYN. The unsaturated double bond at the uracil group (responsible for the toxicity of CYN) is probably the most reactive site for the SR reaction. Sulfate radicals also attack the tricyclic alkaloid group, leading to the cleavage of the sulfate group and operation of the tricyclic ring. Due to the different reaction pathways of UV/H_2_O_2_ compared to of UV/PMS and UV/PS, several unique by‐products were formed only in SR‐AOPs, e.g., C_14_H_21_N_5_O_8_S, C_13_H_21_N_5_O_7_S, and C_14_H_17_N_5_O_4_. Lower molecular weight by‐products with <10 carbons were also observed. This degradation pathway is shown in **Figure** **5**. Considering similar but different preferential reaction mechanisms between hydroxyl radicals and sulfate radicals, fewer hydroxylation byproducts are likely to be formed in the UV/S_2_O_8_ system in the presence of NOM; thus, lower formation of degradation by‐products (DBPs) be expected.^[^ ^77^ ^]^ Very significant degradation (98.6%) of ATX was achieved with the UV/PMS system; however, the degradation pathway is still not elucidated.^[^ ^64^ ^]^

#### 3.1.2 Degradation Mechanism of MC‐LR through SR‐AOP

**Figure 6:**
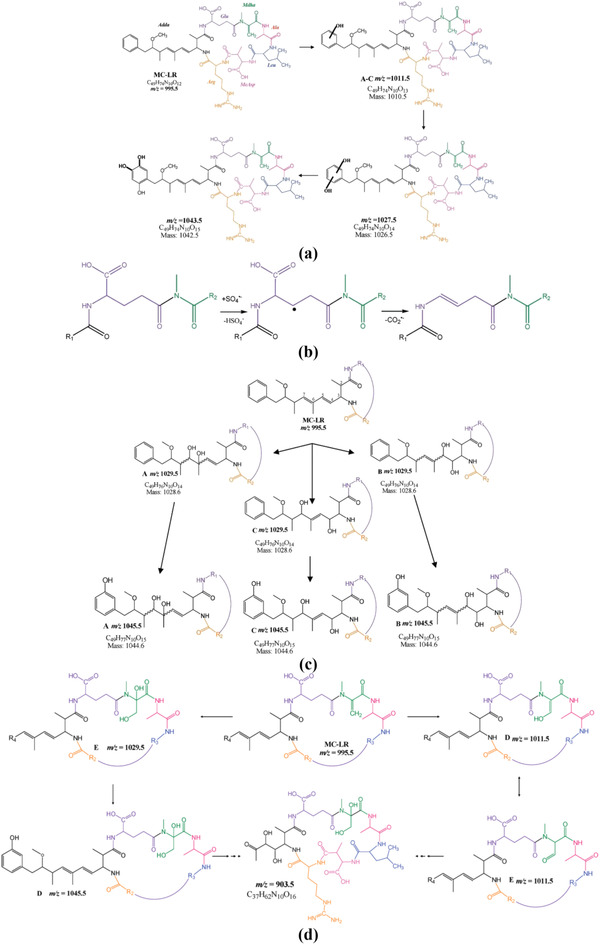
Four different pathways of Microcystin‐LR with SR‐AOP a). Multiple Hydroxylation of MC‐LR with Sulfate Radicals, b). Decarboxylation of Glu Amino Acid with Sulfate Radicals, c). Simultaneous Hydroxylation of the Aromatic Ring and the Diene Bond of MC‐LR with Sulfate Radicals, d). Simultaneous Oxidation of the Cyclic Structure and the Chain of MC‐LR with Sulfate Radicals. Adapted with permission.^[^ ^61^ ^]^ Copyright 2010, American Chemical Society.

Microcystins (MCs) are a group of cyanotoxins that have a cyclic heptapeptide structure and can cause the growth of liver cancer. These cyclic heptapeptides consist of five fixed amino acids and two variable protein amino acids that are unique to each microcystin derivative. Currently, over 60 variants of microcystin have been identified. Their cyclic chemical structure and functional groups contribute to their high stability under extreme conditions and solubility in water. The most toxic and commonly detected derivative of microcystin in water sources is MC‐LR, where the letter L and R refer to leucine and arginine, respectively. During the degradation of MC‐LR by sulfate radicals, four pathways have been identified by Antoniou et al.,^[^ ^61^ ^]^ as shown in **Figure** **6**. The first pathway involves hydroxylation of the benzene ring of the amino acid Adda, followed by single, double, and triple hydroxylation. The second pathway involves hydroxylation of the conjugated double bonds of the Adda chain, leading to hydroxylated intermediates and further conversion by decarboxylation. The third pathway involves oxidative cleavage of the Adda amino acid chain, forming a hydroxylated intermediate. The fourth pathway involves hydroxylation of the unsaturated C‐bond of Mdha, with dominant oxidation mechanisms by substitution and addition. Simultaneous oxidation of the unsaturated carbon bonds of Adda and Mdha was also observed.

Since the cyanotoxins are present in natural waterbodies, the effects of various cations, anions, and natural organic matter (NOM) influence the efficiency of SR‐AOPs. One of these components is bromide ions. Concentrations of bromide in freshwater typically range from trace amounts to about 0.5 mg L^−1^. It has been observed that the UV/persulfate treatment process results in significant conversion of bromide to bromate at relevant UV and PMS/PDS dosages, indicating a potential problem of excessive bromate production when SO_4_^•−^‐based AOPs are used to treat bromide‐containing drinking water and wastewater. NOM can reduce the formation of bromate by reacting with intermediates such as HOBr/OBr^−^ and Br^•^, or SO_4_^•−^. However, this could lead to the formation of bromine‐containing by‐products that may pose health risks, while SO_4_^•−^ reduction could reduce the amount of SO_4_^•−^ available for destroying micropollutants. Thus, unless there is an abundance of NOM to consume the bromine intermediates, SO_4_^•−^‐ induced conversion of bromide to bromate is still expected to play an important role in the destruction of micropollutants at the same SO_4_^•−^ exposure. To prevent the formation of bromate, increasing the pH above 8 may be an option for effective degradation that is not negatively affected by the pH increase.

The sulfate radical can cause the transformation of bromide into the harmful substance bromate. However, the presence of aromatic organic matter can impede this process, resulting in negligible amounts of bromate being formed in SR‐AOPs during routine water treatment.^[^ ^78^ ^]^

## 4 Conclusion

AOPs are designed to eliminate the bioavailable fraction of cyanotoxins that are in direct contact with aquatic organisms or water users, which are usually dissolved in water. However, in untreated raw waters, cyanotoxins are not only dissolved but also stored inside cyanobacterial cells, especially while the bloom is still healthy. The intracellular fraction varies depending on the cyanotoxin type, being higher for MCs and lower for STXs and CYN. Therefore, before applying AOPs, cyanobacteria must be removed to a high extent because the attack of ^•^OH on cyanobacterial cells causes their lysis and releases cyanotoxins into the water.

The effectiveness of drinking water treatment plants (DWTPs) in removing cyanotoxins, with conventional processes such as adsorption on activated carbon and chlorination by oxidation is being relatively effective but also exhibiting significant drawbacks. For instance, some cyanotoxins, such as ATX and STX, remain resistant to these treatments. Additionally, high carbon or chlorine doses and long contact times are typically necessary. Advanced membrane filtration technologies are not always reliable for removing all types of cyanotoxins and require high energy consumption. In this regard, AOPs, which utilize in situ generated hydroxyl radicals, represent the most promising technologies for cyanotoxin removal, given their ability to oxidize the main cyanotoxin families, including MCs, ATX, CYN, and STX. This is in contrast to conventional primary oxidants like chlorine, chlorine dioxide, hydrogen peroxide, ozone, chloramines, and potassium permanganate, which offer limited versatility. An AOP should not only ensure the elimination of cyanotoxins but also prevent the formation of toxic intermediates.

While there is potential for SR‐AOPs to remove organic chemicals and cyanotoxins, there may be certain limitations:

a) Sulfate ions can be produced as a by‐product of sulfate radical‐based AOPs as residual sulfate ions. To comply with legal discharge restrictions or lessen their negative effects on the environment, these residual sulfate ions could need additional treatment or control. An increase in residual cations may be caused by AOPs involving sulfate radicals, such as the production of sodium (Na+) and potassium (K+) ions. Additional treatment procedures can be necessary to lower these cation concentrations in the effluent, depending on the needs for the effluent specifically as well as environmental standards.
b) In comparison to other oxidation processes like hydrogen peroxide (H_2_O_2_), sulfate radical‐based AOPs could be more expensive. This might be because of the price of the reactants used, the necessity for more equipment, or the requirement for specialized catalysts. The usage of persulfate compounds (PMS/PDS) as a source of sulfate radicals in AOPs may necessitate greater dosages of PMS/PDS compared to hydrogen peroxide for the same level of COD removal. This rise in persulfate compound demand may have an effect on the overall operational costs of the treatment procedure.
c) Sulfate radical‐based AOPs may result in the formation of chlorate or bromate molecules when chloride ions (Cl^−^) and bromide ions (Br^−^) are present. It may be required to use monitoring and control measures to stop the creation of these by products or to eliminate them from the treated effluent.
d) Intensification of the toxicity evaluation for SR‐AOPs treated waters, by both acute and chronic toxicity tests are necessary for the determination of toxicity level. Ideally, further studies should be conducted on the toxicity assessment or the determination of toxic by‐products formed in real water matrix.

Unlike conventional AOPs where the main oxidant is ^•^OH, SR‐AOPs primarily target the radical species SO_4_^•−^, which exhibits significant specificity toward substrates. Depending on the activation method and water matrix, other selective oxidants such as _1_O^2^, hydroxyl radicals and high‐valent metals (e.g., Fe(VI)) may also be formed. It is noteworthy that some persulfate‐AOPs do not even involve radical species, especially when heterogeneous catalysts are used. This unconventional approach provides an opportunity to explore niche applications by tailoring the oxidants and oxidation mechanisms to suit the specific water matrix and persulfate activation method, which is a departure from traditional AOPs. In essence, SO_4_^•−^ typically exhibits one or two orders of magnitude smaller second‐order rate constants than ^•^OH, but it outperforms nonradical oxidants, such as O_3_ and ^1^O_2_, regardless of substrate type. It can be inferred that SR‐AOPs provide an appealing treatment choice for eliminating cyanotoxins due to their ability to minimize the loss of oxidizing potential caused by the scavenging of oxidants by natural components present in the water. This is a common issue encountered in AOPs based on ^•^OH radicals. SR‐AOPs can be activated using various methods, including heat, UV radiation, and transition metal catalysts, providing greater flexibility and optimization in treatment design. This allows for the tailoring of SR‐AOPs to the specific water matrix and cyanotoxin type, improving the efficiency and selectivity of the process. Overall, SR‐AOPs offer several advantages for the degradation of cyanotoxins, including selective targeting, lower reactivity with background water constituents, and greater flexibility in treatment design.

## Conflict of Interest

The authors declare no conflict of interest.
